# Reoperative minimal invasive off-pump coronary artery bypass graft in early left main stem stenosis following Modified Bentall procedure

**DOI:** 10.1186/s13019-024-03121-y

**Published:** 2024-12-19

**Authors:** Anupama Barua, Lucy Cosbey, Avishek Samaddar, Ravish Jeeji, Richard Warwick, Lognathen Balacumaraswami

**Affiliations:** 1 Department of Cardiothoracic Surgery, University Hospitals NHS North Midlands NHS Trust, Stoke-on-Trent, UK; 2 Department of Radiology, University Hospitals NHS North Midlands NHS Trust, Stoke-on-Trent, UK; 3 Department of Cardiothoracic Anaesthesia, University Hospitals NHS North Midlands NHS Trust, Stoke-on-Trent, UK; 4 Department of Cardiothoracic Surgery, Royal Stoke Hospital, Stoke-on-Trent, UK

## Abstract

Six months following modified Bentall procedure a patient presented with angina and acute ST depression. CT coronary angiogram revealed severe narrowing of the left main coronary artery. Minimal invasive off pump coronary artery bypass grafting avoided complex reoperative surgery and delivered an excellent clinical outcome.

## Clinical summary

**Fig. 1:**
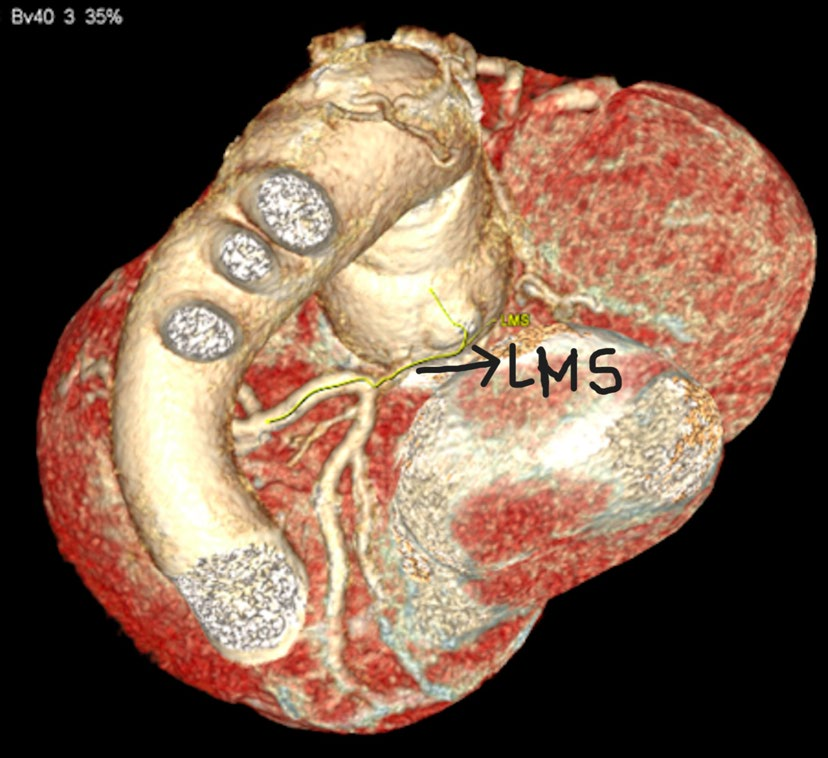
CT demonstrating the narrowing of the left main coronary artery

A 34-year-old gentleman underwent emergency Modified Bentall procedure (25 mm On-X Valsalva composite graft) for treatment of an acutely dilated 7 cm aortic root and severe aortic regurgitation. He made an uneventful recovery. His routine surveillance CT scan demonstrated narrowing of an elongated left main stem (LMS) coronary artery (Fig. 1) and transthoracic echocardiography revealed a normal mechanical AVR functioning well. Left ventricle was moderately dilated and the interventricular septal wall appeared discordant and severely hypokinetic with ejection fraction of 35%. The genetic studies were reported as heterozygous for the pathogenic variant in the Elastin (ELN) gene.

**Fig. 2:**
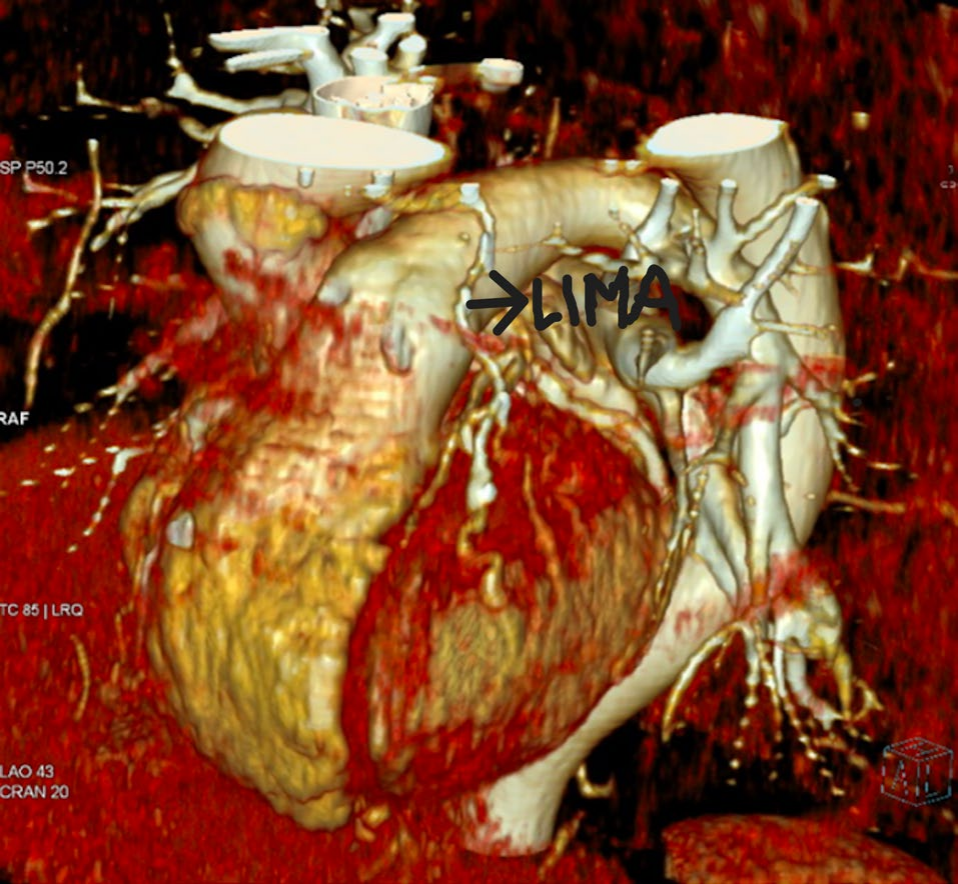
CT demonstrating the patency of LIMA

Six months later, he attended the local hospital with severe chest pain. Angina was not relieved by nitrates and troponin level was within the range. ECG revealed widespread ST depression in V_5_-V_6_ and T wave depression in V_3_-V_4_. He underwent emergency minimal invasive off pump coronary artery bypass grafting (Mini-OPCABG) to avoid the complexity of early reoperative surgery. A 6 cm subareolar incision was performed. The 5th intercostal space was entered and particular care was taken to avoid injury to cartilage and ribs. Skeletonised left internal mammary artery (LIMA) was harvested using the Medtronic Thoratrak™ Retractor system. Anterolateral approach provided excellent access with minimal adhesions in the reoperative setting. LIMA was anastomosed to the readily accessible diagonal artery (1.75 mm) which was larger calibre than native left anterior descending artery without any disease in the entire left coronaty system beyond the narrowed LMS. The graft supplies the entire left system. The patient was discharged on fourth post-operative day uneventfully and continues to remain well and completely angina free one year following surgery. Follow up CT scan in a year showed excellent patency of LIMA graft (Fig. 2).

## Comment

Modified Bentall procedure is a gold standard technique for aortic root dilatation supported by long term data [1]. However, proximal coronary artery complications are well documented. Proximal coronary aneurysms are common in the late postoperative period [2]. The incidence may be as high as 50% in younger patients with Marfan’s syndrome [3]. Stenosis or narrowing in the proximity of neo coronary ostium is not uncommon. Turbulent blood flow between the Dacron graft and the native coronary artery may contribute to the stenosis. Tension free anastomosis respecting the anatomical orientation is a prerequisite for laminar blood flow.

The incidence of coronary artery stenosis after aortic root replacement reported in literature is 0.3 − 5% [4]. Various mechanisms contribute to this sequel such as a stretched graft, mechanical injury during manipulation of cardioplegia cannula, high infusion pressure during transfusion of cardioplegia solution and inadequate mobilisation the left main stem during the Bentall procedure. Inappropriate tissue handling results in inflammatory reaction and stenosis and fibrous tissue formation in the proximity of the coronary ostia. Surgical correction is the definitive treatment.

Resternotomy is associated with a substantial risk of mortality and morbidity and this is most pronounced within 12 months of primary surgery. Mini-OPCABG is an ideal approach in suitable cases [5, 6]. It eliminates the risk of full redo sternotomy which can be potentially hazardous. Avoidance of extensive adhesiolysis, aortic manipulation and cardiopulmonary bypass is beneficial [7, 8]. This case illustrates the advantage of Mini-OPCABG following modified Bentall Procedure. It allows ready access to the target requiring minimal dissection around the heart.

The patient recovered uneventfully without any complications and was discharged home on the 4th post operative day. Our strategy was a well planned Mini-OPCAB pathway for early recovery. This involved.

1. Pre-emptive analgesia with local intercostal block at incision site.
2. ET tube with single lumen blocker.
3. Short acting anaesthetic protocol.
4. Paravertebral block after completion of the procedure.
5. On table extubation.
6. Early mobilisation and physiotherapy.
7. Early discharge.

The patient benefits from expedited recovery, minimal blood loss, early mobilisation and early discharge at four days with return to routine activities within 3 weeks of surgery.

## Data Availability

No datasets were generated or analysed during the current study.
